# Plasma metabolomics in male primary and functional hypogonadism

**DOI:** 10.3389/fendo.2023.1165741

**Published:** 2023-05-26

**Authors:** Giuseppe Grande, Luca De Toni, Andrea Garolla, Domenico Milardi, Alberto Ferlin

**Affiliations:** ^1^ Unit of Andrology and Reproductive Medicine, Department of Medicine, University of Padova, Padova, Italy; ^2^ Division of Endocrinology, Fondazione Policlinico Universitario “Agostino Gemelli” Scientific Hospitalization and Treatment Institute (IRCCS), Rome, Italy

**Keywords:** testosterone, hypogonadism, proteomics, metabolomics, markers

## Abstract

Metabolomics proposes to unveil the molecular machinery involved in each specific disease by the comprehensive analysis of low-molecular-weight metabolites in a biological sample. This narrative mini-review analyzes previous studies applying ultra-high-performance liquid chromatography–high-resolution mass spectrometry (HRMS)-based metabolomics to highlight different metabolic pathways involved in male hypogonadism and testosterone replacement therapy, both in the case of insulin-sensitive patients with primary hypogonadism and in the case of insulin-resistant patients with functional hypogonadism. In functional hypogonadism, metabolomics revealed that different biochemical pathways are affected. In detail, glycolysis is the most important biochemical process involved in these patients. Glucose metabolism is fueled by amino acid degradation, and gluconeogenesis is widely stimulated. Some important pathways, including glycerol, are compromised. Furthermore, mitochondrial electron transport is influenced, namely, by a decrease in ATP production. On the contrary, beta-oxidation of short- and medium-chain fatty acids does not represent an energy source in hypogonadal patients. Both lactate and acetyl-CoA are converted into ketone bodies, which increased immensely. However, carnosine and β-alanine are greatly reduced. These metabolic changes are associated with increased fatigue and mental confusion. After testosterone replacement therapy, a complete restoration is achieved for only a part of the metabolites. It is of note that only in patients with functional hypogonadism treated with testosterone are ketone bodies produced at high levels, so the symptoms sometimes reported by these patients after the beginning of the therapy (difficulty in concentrating, depressed mood, brain fog, and memory impairment) might represent a specific “keto flu-like” syndrome, related to the metabolic ketonic state.

## 1 Male functional hypogonadism and metabolomics

Male hypogonadism derives from the deficiency of the testis to secrete physiological concentrations of testosterone (T) (T deficiency) and/or a normal amount of spermatozoa as a consequence of a disease of the hypothalamic–pituitary–testicular (HPT) axis (1). According to the site primarily involved, we can define primary hypogonadism (hypergonadotropic hypogonadism) as caused by a primitive testis disease or secondary hypogonadism (hypogonadotropic hypogonadism (HH)) due to a disorder compromising the pituitary function. In primary hypogonadism, low or normal T levels and high gonadotropin levels are observed, while in secondary hypogonadism, patients have low T levels associated with normal or reduced follicle-stimulating hormone (FSH) and luteinizing hormone (LH) levels.

Male hypogonadism may be classified, moreover, according to the onset of the symptoms (late-onset hypogonadism) or on the basis of the potential reversibility of the disorder. The term “organic hypogonadism” is identified as a non-reversible form of hypogonadism due to a congenital or acquired defect in the HPT axis (2). Differently, “functional hypogonadism” defines a potentially reversible condition. The highest prevalence of functional hypogonadism has been reported among middle-aged or older men (>40–50 years). An association with comorbid illnesses—frequently obesity—has been reported. It is associated with a lower reduction of T concentrations with respect to organic forms (3, 4). Therefore, in middle-aged or older men, androgen deficiency might occur without intrinsic structural hypothalamic–pituitary–testis axis diseases or specific diseases causing the suppression of the HPT axis. This functional hypogonadism recognizes its major cause of metabolic alterations, including obesity and type 2 diabetes mellitus (4). Longitudinal data derived from the European Male Ageing Study (EMAS) incontrovertibly demonstrated that both obesity and weight gain are associated with an increased risk to develop functional secondary hypogonadism. In subjects who lost weight, conversely, T levels are upregulated so that recovery from secondary hypogonadism is frequent (5, 6). The molecular machinery involved in functional hypogonadism has not been completely clarified. Nevertheless, it is believable that metabolic alterations and the inflammatory condition due to obesity may interfere in direct and indirect ways with the release of T or gonadotropins at different levels (7, 8).

However, T has an important influence both on body fat regulation and on the maintenance of normal bone and muscle mass (9). Testosterone plays in fact a pivotal role in glucose homoeostasis and in controlling lipid metabolism (10). Furthermore, male hypogonadism is associated with metabolic dysfunction through different mechanisms (11–13). In detail, male hypogonadism may develop in normoinsulinemic patients (insulin-sensitive (IS) patients). However, over time, an increase in blood insulin concentration is observed, causing metabolic and clinical complications. T deficiency is, in fact, correlated with an increase in fat mass, a reduction of insulin sensitivity, and glucose tolerance. Furthermore, increased levels of triglycerides (TGs) and free cholesterol and reduced levels of high-density lipoprotein-cholesterol are observed in hypogonadal patients.

The word “-omics” refers to the study of biological systems on a large scale. Metabolomics attempts to unveil the molecular phenotype of a specific disease by the comprehensive analysis of low-molecular-weight metabolites in a biological sample. Metabolites are intermediate products or end-products of cellular metabolism. Metabolomics, therefore, represents the arrival point of the “-omics” cascade (14).

In this narrative mini-review, the role of metabolomics in the field of hypogonadism is discussed with a particular focus on its use in the light of new insight into the pathophysiological role of T in primary and functional hypogonadism.

## 2 Metabolomics reveals the metabolic pathways involved in primary and functional hypogonadism

Metabolomics had helped in revealing the different metabolic pathways involved in both primary and functional hypogonadism and describing how testosterone replacement therapy (TRT) may modify the dysmetabolic pathways.

In 2018, Fanelli and colleagues performed a serum metabolomic analysis by ultra-high-performance liquid chromatography–high-resolution mass spectrometry (HRMS) in 15 patients with male primary hypogonadism and normal insulin sensitivity, compared with 15 normogonadic subjects (15). The pentose phosphate pathway (PPP), Krebs cycle, and β-alanine metabolism were the pathways more frequently impaired in hypogonadal patients. Otherwise, no significant changes in glycolysis have been reported, suggesting that glucose represents the most used biofuel in the muscle, adipose tissue, and liver and that alternative sources are minimally used. Gluconeogenesis is inactive. Furthermore, 3-glycerol is not significantly used to produce triglycerides since no accumulation of dihydroxyacetone was observed. Interestingly, primary hypogonadism induced a significant increase in lactate production. The pentose phosphate pathway was strongly enhanced, suggesting a situation of oxidative stress, as confirmed by the increase in the levels of oxidized glutathione (GSSG). A reduction in acyl-carnitine was reported in patients with hypogonadism, indicating the downregulation of fatty acid burning by β-oxidation. Cholesterol production slightly increased according to previous evidence about the effect of low T levels in promoting cholesterol mobilization from the liver (16). The study demonstrated, moreover, that in hypogonadism, amino acids are not used to produce energy, although glutamine and aspartate were used for glutaminolysis more than in controls. Moreover, the authors reported a reduction in the metabolism of proline and lysine, which may be linked to a reduction in the production of collagen, thus explaining the lower lean body mass observed in hypogonadal patients. The reduction in collagen and subsequently in lean body mass, in association with a lower carnosine formation, may contribute to the fatigue during isokinetic and isometric exercise reported in these patients.

Furthermore, the same authors analyzed the metabolomic profiles of patients with primary hypogonadism after 60 days of administration of TRT (17). After the restoration of testosterone levels, not all metabolic pathways were restored. In detail, a reactivated and accelerated glycolysis (as demonstrated by lactate increase) represented the major source of energy. Active insulin permits in fact the triggering of the Cori cycle in order to furnish energy. Furthermore, it prevents the development of ketone bodies. The level of acetyl-CoA increased significantly. A fraction of acetyl-CoA was converted to mevalonic acid and then involved in cholesterol synthesis, although cholesterol levels in patients are not significantly increased, confirming the hypothesis that a longer period of treatment is needed to restore all lipid metabolisms. An increase in fatty acid production from acetyl-CoA has been reported. After testosterone treatment, moreover, a reduction in the levels of acetyl-carnitine was reported. Acetyl-carnitine is fundamental in transporting fatty acids from the cytoplasm to mitochondria for β-oxidation. Consequently, more triglycerides were produced. Decreased free carnitine levels were also recorded, so the authors suggested integrating carnitine in association with TRT in these patients. Finally, a decrease in GSSG was observed, confirming that TRT reduces the condition of oxidative stress. TRT was, moreover, associated with an increase in proline and lysine, suggesting a positive influence of T in the synthesis of collagen fibers and therefore in the reduction of bone loss and bone degradation.

Similar studies have been then performed in patients with functional hypogonadism associated with obesity and insulin resistance (IR). In hypogonadal patients with insulin resistance, metabolomics showed that 38 biochemical pathways are compromised. In detail, glycolysis was the most consistently altered biochemical process in IR patients. Glucose metabolism was fueled by amino acid degradation, and gluconeogenesis was strongly activated. Some important pathways, including glycerol, are compromised. Furthermore, mitochondrial electron transport is influenced, namely, by a decrease in ATP production. On the contrary, beta-oxidation of short and medium-chain fatty acids does not represent an energy source in hypogonadal patients. Both lactate and acetyl-CoA are converted into ketone bodies, which increased immensely. However, carnosine and β-alanine are greatly reduced. These metabolic changes are associated with increased fatigue and mental confusion (18).

We further studied metabolomic profiles in IR hypogonadal patients after 60 days of TRT (19). As for insulin-sensitive hypogonadal patients, a complete restoration was obtained only for some of the metabolites. After TRT, a slight improvement in glycolysis was recorded, so lactate was significantly higher after TRT. Gluconeogenesis was stopped upon TRT. As a result of the increased levels of lactate, lactate dehydrogenase is inhibited with a consequently reduced conversion of pyruvate to lactate. Pyruvate is therefore transformed into acetyl-CoA by PDH enzymes, which are not downregulated by insulin. This evidence may clarify the significant increase observed in acetyl-CoA levels. In addition, the increase in acetyl-CoA may derive from the increased degradation of leucine/isoleucine too. Thus, TRT significantly increased the production of acetyl-CoA. Furthermore, acetyl-CoA is preferentially transformed into the ketone bodies acetoacetate and 3-hydroxybutyrate so that TRT induces in IR hypogonadal patients the production of ketone bodies. It is important to underline that ketone bodies are increased after TRT only in hypogonadal patients with IR. On the contrary, TRT is not associated with ketone body (KB) increase in insulin-sensitive patients with hypogonadism, underlining the link between testosterone and insulin resistance for KB production in male hypogonadism. This metabolic ketonic state might explain some symptoms that are sometimes reported by patients after the beginning of TRT, such as difficulty in concentrating, depressed mood, brain fog, and memory impairment. Therefore, the existence of IR hypogonadal patients receiving TRT of a specific “keto flu-like” syndrome has been proposed.

The major metabolic differences between patients with primary and functional hypogonadism, before and after TRT, are summarized in **Table 1**.

**Table 1:** Major differences in metabolic pathways in primary and functional hypogonadism, before and after TRT.

	Primary hypogonadism		Functional hypogonadism	
Metabolic pathway	Before TRT	After TRT	Before TRT	After TRT
Glycolysis	reduced	increased	**highly reduced**	increased
Gluconeogenesis	inactive	inactive	**increased**	reduced
Aminoacid cathabolism	inactive		**increased**	reduced
Ketone bodies formation	inactive		increased	**highly** **increased**

In bold values the major differences in functional hypogonadism vs primary hypogonadism.

Further studies are needed to confirm this evidence and to investigate the putative role of lifestyle and dietary modifications on the different metabolic pathways in hypogonadal patients, including the supplementation with carnitine in patients with primary hypogonadism, and in association with TRT, as suggested by metabolomic studies or the effect of diet and insulin‐sensitizing agents on metabolic profiles in patients with functional hypogonadism.

Although metabolomics provided interesting information about the metabolic pathways compromised in male functional and primary hypogonadism, the technical platform has several major limitations. The results of MS-based metabolomics are in fact highly dependent on the type of mass spectrometer, as well as the sample processing protocol and data processing pipeline used. Furthermore, metabolomics can only show one picture of a system that dynamically transits among different states, which, moreover, depends on phenotype, dietary habits, ethnicity, or other concurrent diseases. If some of these biases may be solved by a proper design of the study, comparing similar populations differing—as far as possible—only for the studied variable (i.e., functional *vs.* primary hypogonadism), other questions can be solved only in a higher perspective of multi-omics. Since each -omic science explores a specific layer and gives one specific picture of a complex system, only integrating multiple omics information may help in improving the understanding of a particular knowledge domain. Further studies are therefore needed to integrate genomic (20), proteomic (21), and metabolomic data in male hypogonadism to portray a systematic view at the molecular level on this topic.

## 3 Conclusions

Ultra-high-performance liquid chromatography–HRMS-based metabolomics has been used to solve some of the pathophysiological questions related to male hypogonadism and identify new clinical markers of male hypogonadism. In detail, serum metabolomics highlighted different metabolic pathways involved in male hypogonadism and TRT, both in the case of insulin-sensitive patients with primary hypogonadism and in the case of IR patients with functional hypogonadism. From this review, it is clear that the number of studies is still too low to draw conclusions, and further research is needed to gain more insight into this fascinating topic.

## Author contributions

GG, AF and AG contributed to the conception and design of the study. GG, DM and LT performed the bibliographical research. GG wrote the first draft of the manuscript. All authors contributed to the manuscript revision, and read and approved the submitted version.
